# Microbial metabolites and heart failure: Friends or enemies?

**DOI:** 10.3389/fmicb.2022.956516

**Published:** 2022-08-15

**Authors:** Xiaofeng Lu, Jingjing Liu, Bing Zhou, Shuwei Wang, Zhifang Liu, Fuyang Mei, Junxiang Luo, Yong Cui

**Affiliations:** ^1^Department of Cardiovascular Surgery, Heart Center, Zhejiang Provincial People’s Hospital (Affiliated People’s Hospital, Hangzhou Medical College), Hangzhou, China; ^2^Bengbu Medical College, Bengbu, China; ^3^Department of Critical Care Medicine, Lishui Hospital of Traditional Chinese Medicine, Lishui, China

**Keywords:** heart failure, microbial metabolites, trimethylamine N-oxide (TAMO), diet, therapy

## Abstract

Heart failure (HF), a global health issue characterized by structural or functional cardiac dysfunction, which was found to be associated with the gut microbiome recently. Although multiple studies suggested that the gut microbiome may have an impact on the development of cardiovascular diseases, the underlying mechanism of the gut microbiome in HF remains unclear. The study of metabolites from gut microbiota influenced by dietary nutrition uptake suggested that gut microbiota may affect the process of HF. However, on the basis of the microbiota’s complicated roles and their interactions with metabolites, studies of microbial metabolites in HF had rarely been described so far. In this review, we focused on dietary nutrition-related factors that were involved in the development and progression of HF, such as trimethylamine N-oxide (TMAO), short-chain fatty acids (SCFAs), and bile acids (BAs), to summarize their advances and several potential targets in HF. From a therapeutic standpoint, we discussed microbial metabolites as a potential strategy and their applications in HF as well.

## Graphical Abstract

Microbial metabolites that impact heart failure and correlated therapeutic strategies. 
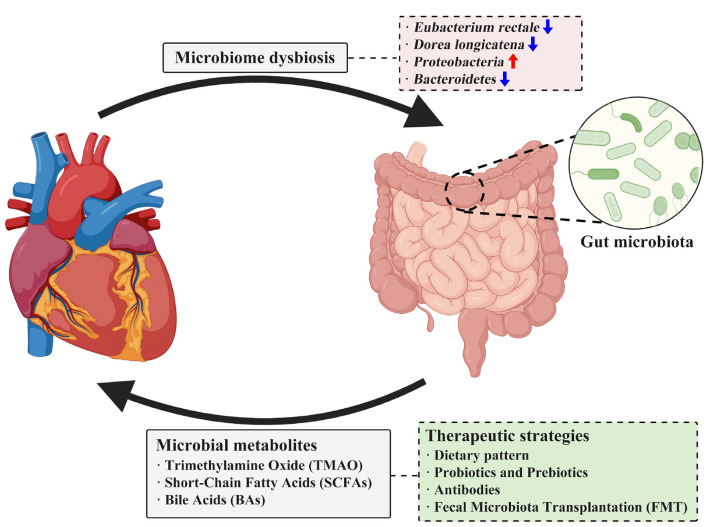

## Introduction

Heart failure (HF) is a multifactorial and systemic syndrome with high morbidity and mortality worldwide (Sinnenberg and Givertz, 2020). Although great advances have been made due to the development and achievement in cardiovascular medications and vascular intervention, the incidence of HF is still increasing and is estimated at around 6 cases per 1,000 persons for the last decades in some western countries (DeVore et al., 2021; Seferović et al., 2021). Factors like age, gender, dietary structure, and metabolic diseases are demonstrated to be the risk factors in HF according to the 2021 European Society of Cardiology (ESC) Guidelines for the diagnosis and treatment of HF (Heidenreich et al., 2022). In the past few years, researchers have shown that the interactions and communications between the translocation of the bacteria from gut to blood and their subsequent pathogenic responses elicited in HF (Fromentin et al., 2022),

however, the underlying mechanisms of how gut microbiota affect the development of HF remain largely unknown.

Human digestive tracts harbor more than 100 trillion microbes that may contribute to several physiological processes in the host (Brito, 2021; Alberdi et al., 2022). Under physiological context, gut microbiota collaborates with the host to help produce energy, synthesize proteins, and absorb nutrients, as well as maintain gut homeostasis (Alwan and Di Meglio, 2021; Le Bras, 2022; Trevelline and Kohl, 2022). These functions confer gut microbiota on the significance in the development of HF. Yet, when it comes to pathological context, gut microbiota dysbiosis may allow endotoxin (e.g., lipopolysaccharide, LPS) produced by gram-negative bacteria translocated from gut to the blood circulation, which subsequently results in the development of cardiomyopathy (Brainin et al., 2021; Drozd and Cubbon, 2021). The study of metabolites produced by gut microbiota that affect their hosts confirmed this hypothesis. Zhang et al. (2021) pointed out that the trimethylamine N-oxide (TMAO), a newly identified microbial metabolite, may contribute to the occurrence and progression of both acute and chronic HF. Further, they found that TMAO was an early predictor that distinguished individuals from other diseases at the risk of cardiac symptoms. This study led to the promise that gut microbiota may not only be a biomarker but also a therapeutic target in HF. On the basis of these results and the corresponding advances achieved in the past few years, we then want to discuss the communications between microbial metabolites and their roles in the development and progression of HF, and the potential therapeutic strategies based on those interactions are reviewed as well.

## Gut microbiome dysbiosis in heart failure

The gut microbiome is a complicated and dynamic system inside the gastrointestinal tract (Kuziel and Rakoff-Nahoum, 2022). The structure of the gut microbiota depends on age, diet, antibiotics, and digestive diseases in humans (Grieneisen et al., 2021). The gut microbiota in healthy individuals belongs to seven major phyla (bacteroidetes, firmicutes, proteobacteria, fusobacteria, verrucomicrobia, cyanobacteria, and actinobacteria) (Adak and Khan, 2019). However, in HF patients, the structure and abundance of gut microbiota altered significantly compared to healthy controls, indicating the potential correlations between the gut microbiome and HF (Spehlmann et al., 2022). Low levels of cardiac output and sympathetic vasoconstriction that result in intestinal ischemia are the major causes of microbial dysbiosis in HF patients (Verbrugge et al., 2013). Intestinal ischemia and hypoxia lead to necrosis of villi cells, which are the principal constituents of the intestinal barrier. Moreover, ischemia can cause intestinal tissue congestion, increasing barrier permeability and thus allowing more bacterial toxins to leak into the circulation.

Heart failure patients are found to have more pathogenic bacterial and less probiotics (Wang et al., 2021). Early in 2017, Kamo et al. (2017) investigated the microbiome of HF patients by 16S rRNA metagenomic analysis and found that *Eubacterium rectale* and *Dorea longicatena* were decreased in HF patients. Moreover, they also found that the composition of gut microbiota altered with ages in HF patients, older patients tended to harbor more *Proteobacteria* but less *Bacteroidetes*. Another study conducted by Kummen et al. (2018) also analyzed the gut microbiota in HF patients and found that chronic HF patients are more likely to have more *Proteobacteria* and less *Bacteroides* as well. Moreover, some specific pathogenic bacteria such as *Shigella*, *Campylobacter*, and *Candida* were also found to be correlated with the severity of HF and even be regarded as potential prognostic indicators in HF (Yuzefpolskaya et al., 2020). The structure of gut microbiota is also different in patients with different subtypes of HF. Microbial structure of heart failure with preserved ejection fraction (HFpEF) patients was demonstrated to be with an increase of *Enterococcus* while a decrease of *Butyricicoccus* in fecal samples (Huang et al., 2021). Meanwhile, studies have also revealed that microbial dysbiosis could induce chronic inflammation and immune dysregulation and ultimately aggravate heart failure with reduced ejection fraction (HFrEF) (Madan and Mehra, 2020). This characteristic in HF patients makes microbiota possible in diagnosing or treatment of HF.

On the basis of these findings, it is possible that HF could be treated by diets or drugs through the gut microbiome. Intake of dietary fiber is related to the incidence of HF. Kaye et al. (2020) found that a high-fiber diet increased the abundance of acetate-producing bacteria and suppresses the development of cardiovascular diseases subsequently. Similarly, Marques et al. (2017) also found that diets with high fiber or acetate could prevent hypertension and HF *via* modulating gut microbiota. Moreover, anti-inflammatory diets like vegetables, fruit, and nuts are able to reduce HF incidence in cigarette addicted population by regulating gut microbiota (Kaluza et al., 2020). This study followed more than 70 thousand people for 14.9 years and found that intake of anti-inflammatory food was negatively correlated to HF incidence because anti-inflammatory diets could partially inhibit those oxidative stress and cell death caused by smoking, as well as regulate and maintain gut microbial homeostasis. Moreover, drugs such as spermidine are demonstrated to improve cardiac function by regulating microbial abundance and diversity (Chen et al., 2021). Spermidine intake could increase the percentage of *Muribaculaceae* in HF mice while its antagonist reduced the ratio of firmicutes/bacteroidetes, which is a biomarker of microbial dysbiosis and is associated with cardiac function. Taken together, these observations indicate the possibilities of a new strategy for microbiota by regulating dietary nutrition and probiotics, which will be an alternative in the treatment of HF in the future.

## The microbial metabolites associated with heart failure

### Trimethylamine N-oxide

**FIGURE 1:**
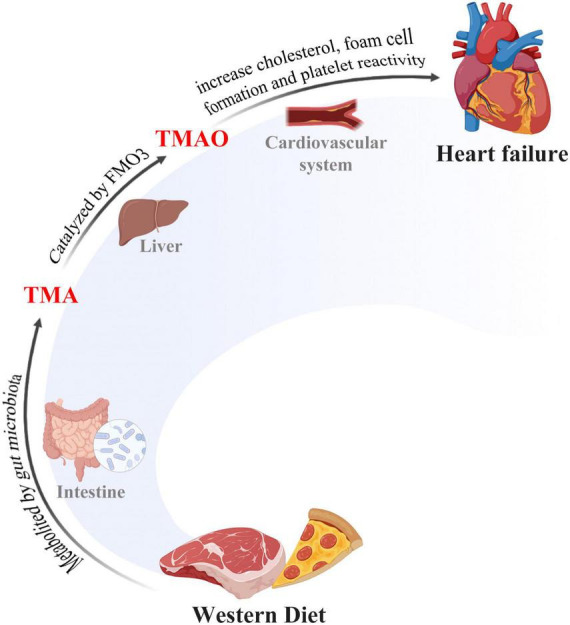
The gut-microbiome-heart axis. Western diet was metabolized to TMA by gut microbiota and then TMA was converted to TMAO in liver tissues. TMAO accumulation triggered cholesterol in many pathological processes, including transportation and foam cell formation, thus inducing heart failure.

The trimethylamine N-oxide, an important molecule from the gut microbial metabolite, whose level is determined by daily uptake of red meat, eggs, dairy food, and saltwater fish that contains choline, lecithin, and L-carnitine, is found to be associated with HF (Naghipour et al., 2021; Li X. et al., 2022). These HF-related substances from daily uptake were catabolized to trimethylamine (TMA) by gut microbiota following digestion. The resulting products are then transformed to TMAO by flavin-containing monooxygenase 3 (FMO3) in the liver (Figure 1). Circulating levels of TMAO in the blood have been suggested to have a positive correlation with HF patients (Suzuki et al., 2019; Drapala et al., 2020), demonstrating its risk role in the development of HF. Indeed, Organ et al. showed that mice fed with TMAO have decreased left ventricular ejection fractions (LVEF) along with increased brain natriuretic peptide (BNP) levels, while dietary elimination of TMAO improves cardiac function and protects the ventricle from remodeling in HF mice (Organ et al., 2020). In addition to this finding, Li et al. reported that cardiac hypertrophy could be induced by TMAO in new-born Sprague–Dawley (SD) rats and inhibition of TMAO by antibiotics alleviates cardiac hypertrophy accordingly (Li et al., 2019). Yet, there have been some contradictory reports on TMAO. Videja et al. (2020) reported that TMAO protects right ventricle cardiac functions from damage in rats by mitochondrial energy synthesis by reducing pyruvate metabolism and preserving fatty acid oxidation. In line with this finding, Gawrys-Kopczynska et al. (2020) reported that TMAO reduces the mortality of HF rats by producing diuresis mechanistically. Thus, the specific role of TMAO remains controversial due to its double-side role in HF, and the corresponding mechanisms of microbiota-derived TMAO in HF are required to be further explored in different contexts (Table 1).

**TABLE 1:** Studies on predictive values of TMAO for HF clinical outcomes.

Patients	Clinical outcomes	References
Acute HF patients (*n* = 972)	In-hospital mortality, all-cause mortality and rehospitalization	Suzuki et al., 2016
HFpEF patients (*n* = 146)	Cardiac-caused mortality and hospitalization	Kinugasa et al., 2021
Acute myocardial infarction (AMI)-related HF patients (*n* = 985)	Major adverse cardiac events (MACE), including all-cause death, MI recurrence and rehospitalization	Li N. et al., 2022
Stable HF patients (*n* = 720)	Mortality	Tang et al., 2014
Chronic HF patients (*n* = 112)	Mortality and heart transplantation	Tang et al., 2015
New-onset or progressively worsening HF patients (*n* = 2234)	Mortality and rehospitalization	Suzuki et al., 2019

Notably, in light of those ambiguities, several studies are conducted to investigate the underlying molecular mechanism of TMAO in HF. Collectively, three mechanisms might be involved in the development of HF, namely, inflammation-induced responses, activation of cell signaling pathway, and interactions among cells or tissues. In detail, inflammation-induced responses in endothelial cells by TMAO promote the development of HF (Brunt et al., 2020). TMAO-triggered activation of NLRP3 canonical inflammasome, leads to the release of pro-inflammatory cytokines, such as IL-1β, IL-6, and TNF-α, resulting in endothelial dysfunction that is responsible for vascular calcification (Zhang et al., 2020). Notably, the same results also demonstrated that TMAO promotes the release of those cytokines by activating the NF-κB signaling pathway in atherosclerosis rats (Li et al., 2021). These studies led to the definition of TMAO-induced HF as an inflammatory disease. However, besides, TMAO is also found to aggravate HF by activating the calcium signaling pathway followed by the gain of function in platelets, which is responsible for the ischemic and non-ischemic HF to occur (Fedotcheva et al., 2021). Dysfunction of the calcium signaling pathway caused by TAMO had been found to impair heart function, and subsequently induce T-tubules remodeling (Witkowski et al., 2021). As one of the risk factors in HF, foam cells triggered by TMAO promoted the formation of atherosclerotic plaque (Steinke et al., 2020; Jiang et al., 2021). Recently, a necrotic programmed cell death, pyroptosis, which is characterized by cell swelling, plasma membrane rupture, and release of cytoplasmic contents, was found in endothelial cells (Wu et al., 2020). Upon pyroptosis execution, necrotic endothelial cells triggered potent immune responses that may cause damage to myocardial cells. Recently, TMAO was found to be involved in T-tubule degeneration induced by TMAO *via* translocation of JPH2 in mouse cardiomyocytes. Collectively, TMAO-induced activation of the calcium signaling pathway was shown to be required for HF to occur, yet, the underlying mechanism of TMAO-induced calcium signaling activation and its association with pyroptosis is required to be further explored.

A recent study suggested that TMAO is not only a target but also a potential biomarker in the process of HF. A prospective observational study showed that serum levels of TMAO with its precursor choline and betaine (an oxidative product from choline) were increased in chronic heart failure (CHF) patients. Moreover, the concentration of these metabolites was found to associate with clinical and hemodynamic severity of HF (Papandreou et al., 2021). This was confirmed by a clinical trial, in which a number of 955 chronic HF patients were followed for 7 years, demonstrating the level of TMAO in HF patients as a predictor of HF (Wei et al., 2021). Similarly, another study by Kinugasa et al. (2021) revealed that serum TMAO, choline, and betaine were correlated with left ventricular diastolic function impairment and poor long-term outcomes in HFpEF patients. Notably, TMAO was shown to be a univariate predictor of the death risk of HFpEF patients. However, the sensitivity of TMAO in predicting heart failure with preserved ejection fraction (HFpEF) has differed from heart failure with preserved ejection fraction (HFpEF). Indeed, Berger et al. (2020) found that the predictive value of TMAO in HfrEF patients was better than in HFpEF patients. Accordingly, it is worthwhile to explore the specificity and sensitivity of TMAO in diagnosing HF in the clinic.

### Short-chain fatty acids

As the main metabolites from dietary fibers, short-chain fatty acids (SCFAs), such as carboxylic acids (e.g., acetyl acid, propionic acid, and butyric acid), are demonstrated to be associated with HF. A lack of dietary fiber in the feeding of SPF and germ-free mice is reported to promote cardiac remodeling (Kaye et al., 2020). SCFAs belong to the metabolites that are produced by gut microbes in gastrointestinal tracts, from which SCFAs were absorbed and transported to the blood circulation *via* the portal vein. Interestingly, SCFAs are not only essential to gut homeostasis but also sufficient for the integrity of the epithelial cell barrier to be maintained. SCFAs execute their functions by the activation of G-protein coupled receptors (e.g., GPR41, GPR43, and GPR109A) signaling pathways and downstream transcription factors, followed by the expression of several anti-inflammatory genes accordingly. Notably, SCFAs are also reported to be the inhibition of Th17 and activation of Treg, which lead to the suppression of immune and inflammatory responses.

Recently, Luedde et al. reported that the gut microbiome in HFpEF patients tended to have deficiencies in Ruminococcaceae and Blautia, which were SCFAs producers (Beale et al., 2021). SCFAs were also beneficial to myocardial electrical remodeling for they could partly activate the parasympathetic nerves through the gut-brain axis (Zhou et al., 2021). As for the underlying mechanism, Carley et al. (2021) further revealed that SCFAs were efficient energy sources for HF patients because failing hearts preferred to oxidize SCFA over ketones. Another similar study found that propionic acids could induce vasodilation and thus alleviate heart failure by binding to GPR41, which is very important in regulating lipid metabolism (Muralitharan et al., 2020). Apart from that, SCFAs were also immunomodulators to their hosts. SCFAs such as butyric acid could mediate various immune cells including Tregs, Th1, macrophages, neutrophilic granulocytes, as well as the antigen-presenting cells including dendritic cells and those inflammatory cells, such as neutrophilic granulocytes (Yang et al., 2020). Taken together with all these benefits, SCFAs were hot topics in clinical strategies now, especially in the treatment of HF.

### Bile acid

Bile acid (BA) is a metabolite of bile synthesized by gut microbes, which plays a pivotal role in lipid metabolism (Funabashi et al., 2020). Primary bile acids were firstly synthesized in the liver tissues and then converted to secondary bile in the gastrointestinal tracts with the help of gut microbiota (Winston and Theriot, 2020). BA was important in facilitating gut nutrient absorption, regulating lipid, and energy metabolism, as well as maintaining gut homeostasis (Chiang and Ferrell, 2019). Dietary habits, fasting, and circadian rhythms have impacts on the production and re-absorption of BA (Moszak et al., 2020; Yang and Zhang, 2020). Receptors of BA signaling, such as the farnesoid-X receptor (FXR), are expressed in almost all the cardiovascular cells and are closely related to electrical conduction and cellular mechanics in heart tissues. Therefore, the BA signaling is very important in regulating the physiological process and many heart diseases of the host (Vasavan et al., 2018). Recently, studies have focused on the therapeutic potentials of BA on diseases, especially cardiovascular disorders. A prospective cohort study conducted by Mayerhofer et al. (2017) assessed the levels of both primary and secondary BA in CHF patients and then revealed a significant reduction in the level of primary BA and an increase in secondary BA. Doctor Mayerhofer ascribed these findings to the function of microbiota, because microbial metabolism impacts BA synthesis greatly, especially the secondary BA (Mayerhofer et al., 2017). This work revealed a close correlation of BA and the gut microbiota in regulating myocardial functions, but the underlying mechanism remained unknown. Farnesoid X receptor (FXR) and the G-protein coupled receptor 5 (TGR5) are two important molecules in the BA signaling pathway (Chiang and Ferrell, 2020; Xu et al., 2021). A study conducted by Xia et al. (2018) reported that FXR was a potential therapeutic target to HF patients, because FXR could ameliorate cardiac dysfunction and promote myocardial remodeling by increasing adiponectin. Moreover, knock-out of FXR facilitated the restoration of failing heart through inhibiting apoptosis and fibrosis in cardiocytes (Gao et al., 2018).

## Interventions targeted at microbial metabolites

### Dietary intervention

Dietary interventions are effective strategies for maintaining microbial homeostasis and treating diseases because the structure and diversity of the gut microbiota are closely correlated with dietary habits (Holscher, 2020; Trefflich et al., 2020; Rautmann and de La Serre, 2021). Dietary modifications including reducing salt intake and maintaining water-electrolyte balance, were very helpful to HF treatment (Jayachandran et al., 2020). Dietary Approaches to Stop Hypertension (DASH), a designed dietary approach characterized by a high intake of grains, vegetables, and low intake of fat, was strongly recommended to those individuals at the risk of cardiovascular diseases (Filippou et al., 2020; Wickman et al., 2021). A study that followed up 412 participants revealed that those individuals who followed DASH diets were less likely to develop HF (Juraschek et al., 2021), and DASH diets were an effective non-pharmacologic strategy to prevent HF (Fu et al., 2020). Apart from DASH, Mediterranean diets, characterized by large proportions of fish, fruits, nuts, and relatively low proportions of red meat, fat, and sugar, are also proved to be effective in preventing cardiovascular disease (Barrea et al., 2021; Khan et al., 2021). A randomized clinical trial served 209 participants diagnosed with CHF with Mediterranean diets and found that Mediterranean diets significantly decreased the severity of HF (Tuttolomondo et al., 2020). Hence, adherence to healthy dietary patterns, such as DASH and the Mediterranean diet, may have benefits in preventing HF.

### Probiotic and prebiotic treatment

Probiotics and prebiotics could maintain and restore microbial homeostasis and protect the host from myocardial damage (Wieërs et al., 2019; Collinson et al., 2020). In probiotics, the microorganisms that are reported to be beneficial to health, several types such as *Bifidobacteria*, *Lactobacillus*, *Akkermansia*, and yeasts have been demonstrated to play a protective role in HF (Suez et al., 2019). Recently, a randomized clinical trial, in which 90 participants had been followed up, revealed that the probiotic yogurt was able to reduce the serum oxLDL and suppress the oxidative reaction in HF patients (Pourrajab et al., 2020). Further, another study found that the intestinal barrier was a potential therapeutic target for HF, and a combined probiotic intervention is capable of repairing gut mucosa (Yu et al., 2021). Besides, in an animal model, the cardioprotective effect of probiotics was also identified. Gan et al. (2014) fed HF rats with *Lactobacillus rhamnosus* GR-1, and a suppression of myocardial remodeling was found finally in HF. As a fermentation product that may alter the microbial composition and/or the activity of hosts, prebiotics are shown to have a protective effect on HF as well (Peng et al., 2020). However, a study conducted by Jama et al. (2020) made a controversial claim that although dietary fiber modulates gut microbiota, the prebiotic fiber is unable to override mice’s genetic predisposition to HF. Both probiotics and prebiotics are capable of protecting the host from HF, while the controversial role of prebiotics needs more attention in the future.

### Antibiotic treatment

The antibiotic treatment provides a strategy for restoring the gut microbiome when microbial dysbiosis occurred in HF. On the basis of the correlation between microbial dysbiosis and HF (Gutiérrez-Calabrés et al., 2020; Han et al., 2021), antibiotics, which, as a commonly used drug in clinics, showed the most effective and conventional way to restore the gut microbiome (Ramirez et al., 2020). By using this strategy, Riba et al. (2017) showed that doxycycline, an antibiotic useful for the treatment of a number of infections, not only improved the left ventricular systolic function but also reduced the severity of HF, and thus protected HF rats from cardiomegaly, cardiac remodeling and fibrosis in an isoproterenol-induced HF model in rat. Similarly, doxycycline was also demonstrated to exert these functions by inhibiting mitochondrial fission and depolarization in cardiomyocytes. Infective endocarditis (IE), especially infection with Methicillin-resistant Staphylococcus aureus (MRSA), is also an important risk factor for congestive HF (Fernández-Hidalgo et al., 2018). Martí-Carvajal et al. (2020) have conducted a comparison of different types of antibiotics for IE treatment and found vancomycin was very effective in inhibiting MRSA infection. However, patients with congestive HF sometimes exhibit reduced vancomycin clearance (Shimamoto et al., 2013). Therefore, given the adverse effects that antibiotics may induce in microecological dysfunction and drug resistance in infectious diseases, it is of great importance to balance their clinical efficacy and adverse effects before using antibiotics (de Gunzburg et al., 2018; Palleja et al., 2018).

### Fecal microbiota transplantation

As a promising and fascinating medical intervention, by transferring gut microbiota from healthy donors to recipients (El-Salhy et al., 2020), fecal microbiota transplantation (FMT) helps recipients restore their microbial homeostasis significantly and was proved to be very effective in *Clostridium difficile* infection (Aira et al., 2021; Fujimoto et al., 2021). Apart from bowel diseases, FMT is also a promising strategy in many cardiac disorders. Zhang et al. (2022) revealed that colonization with youthful gut microbes could recover the microbial homeostasis in aged rats, thus preventing aged-related atrial fibrillation. Similarly, Chen et al. (2020) also found that transplantation of microbes to germ-free mice could restore the arterial remodeling and promote neointimal hyperplasia. However, it is still unclear that whether FMT could treat HF patients in the clinic and the feasibility of FMT application is also needed to be further elucidated in the future.

### Trimethylamine N-oxide inhibitors

Apart from those interventions targeted at gut microbes, drugs targeting metabolic signaling are promising as well. Wang et al. found an analog of choline that could inhibit the key enzyme cutC/D during TMAO formation and was effective in preventing cardiometabolic diseases (Wang et al., 2015). Similarly, Roberts et al. also synthesized two inhibitors, which could inactivate cutC/D permanently, and found that these two inhibitors were effective in reducing the level of TMAO in mice (Roberts et al., 2018). Based on these findings, developing and using novel drugs that targeting at TMAO signaling, are potential strategies in HF treatment.

## Conclusion

Over the past few years, more and more researchers are participating and interested in the field of gut microbiota and cardiovascular systems, especially in the interactions and communications between gut microbiota and cardiovascular diseases. Yet, the specific factors that may responsible for the HF to occur are still confusing. Although emerging studies revealed that gut microbiota may have an impact on the development of cardiovascular disease by microbiota diversity and metabolites, direct evidence on their communications and consequences on HF is still unclear. Current studies showed the evidence in lab and epidemiology that gut microbiota is found to be associated with HF. However, its communications and cross-talk pattern is needed to be elucidated in the future. On the basis of its metabolism nature, gut microbiota metabolite was found to execute their functions by TMAO and SCFAs in HF. Although a substantial number of metabolites from gut microbiota had been investigated in HF, TMAO as a primary metabolite is thought to be required in the development and progression of HF. In line with this rationale, recent studies showed that a diet containing more fibers is associated with higher TMAO levels, suggesting the protectie effects of TMAO in cardiovascular disease. These studies led to the assumption that targeting TMAO, SCFAs, and gut microbiota, as a new strategy in the treatment of HF, will make sense in the future. It is therefore fascinating and promising to explore the role of microbial metabolites in HF, and this will help us to understand the mechanism of gut microbiota on HF and to facilitate the development of optimized therapeutic strategies in HF.

## Author contributions

XL and JjL drafted this manuscript. BZ, ZL, FM, and SW collected the references. YC and JL revised the manuscript. All authors read and approved the final manuscript.
